# Coupling amplified DNA from flow-sorted chromosomes to high-density SNP mapping in barley

**DOI:** 10.1186/1471-2164-9-294

**Published:** 2008-06-19

**Authors:** Hana Šimková, Jan T Svensson, Pascal Condamine, Eva Hřibová, Pavla Suchánková, Prasanna R Bhat, Jan Bartoš, Jan Šafář, Timothy J Close, Jaroslav Doležel

**Affiliations:** 1 Laboratory of Molecular Cytogenetics and Cytometry, Institute of Experimental Botany, Sokolovská 6, CZ-77200 Olomouc, Czech Republic; 2 Department of Cell Biology and Genetics, Palacký University, Šlechtitelů 11, CZ-78371 Olomouc, Czech Republic; 3 Department of Botany and Plant Sciences, University of California, Riverside, CA-92521-0124, USA; 4 VKR Center of Excellence Pro-Active Plants, Department of Plant Biology and Biotechnology, University of Copenhagen, DK-1871 Frederiksberg C, Denmark

## Abstract

**Background:**

Flow cytometry facilitates sorting of single chromosomes and chromosome arms which can be used for targeted genome analysis. However, the recovery of microgram amounts of DNA needed for some assays requires sorting of millions of chromosomes which is laborious and time consuming. Yet, many genomic applications such as development of genetic maps or physical mapping do not require large DNA fragments. In such cases time-consuming *de novo *sorting can be minimized by utilizing whole-genome amplification.

**Results:**

Here we report a protocol optimized in barley including amplification of DNA from only ten thousand chromosomes, which can be isolated in less than one hour. Flow-sorted chromosomes were treated with proteinase K and amplified using Phi29 multiple displacement amplification (MDA). Overnight amplification in a 20-microlitre reaction produced 3.7 – 5.7 micrograms DNA with a majority of products between 5 and 30 kb. To determine the purity of sorted fractions and potential amplification bias we used quantitative PCR for specific genes on each chromosome. To extend the analysis to a whole genome level we performed an oligonucleotide pool assay (OPA) for interrogation of 1524 loci, of which 1153 loci had known genetic map positions. Analysis of unamplified genomic DNA of barley cv. Akcent using this OPA resulted in 1426 markers with present calls. Comparison with three replicates of amplified genomic DNA revealed >99% concordance. DNA samples from amplified chromosome 1H and a fraction containing chromosomes 2H – 7H were examined. In addition to loci with known map positions, 349 loci with unknown map positions were included. Based on this analysis 40 new loci were mapped to 1H.

**Conclusion:**

The results indicate a significant potential of using this approach for physical mapping. Moreover, the study showed that multiple displacement amplification of flow-sorted chromosomes is highly efficient and representative which considerably expands the potential of chromosome flow sorting in plant genomics.

## Background

Advances in sequencing technologies facilitate rapid progress in understanding plant genome structure, function and evolution. However, the majority of sequencing efforts have targeted plant species with relatively small genomes, typically less than 700 Mbp (for example see [1]). But many plants and important crops, including major cereals such as barley, wheat and rye have genomes that are many time larger [2]. Different strategies have been proposed to tackle these genomes, including reduced-representation sequencing (reviewed by [3]) or the use of ancestral or taxonomically closely related species with smaller genomes [4,5].

We have been pursuing another strategy that is based on our ability to prepare suspensions of intact mitotic chromosomes and to sort individual chromosomes and chromosome arms using flow cytometry [6]. Genome analysis can be simplified by dissecting a large genome into these smaller parts, in some species representing only a few percent of the whole genome, as is the case of wheat [7,8]. Chromosome sorting has been reported in at least seventeen plant species, including major legumes and cereals [6]. Flow-sorted chromosomes have been used in variety of studies, including targeted development of markers for specific genome regions [9-11], physical mapping of DNA sequences using PCR [12,13] and localization of DNA sequences to chromosomes using FISH and PRINS [14-16]. The most attractive application has been the construction of chromosome- and chromosome arm-specific BAC libraries [17-19]. Their availability greatly facilitates development of physical contig maps [20] and positional gene cloning [21] in species with complex genomes.

The construction of BAC libraries requires microgram amounts of high molecular weight DNA. In order to obtain this amount of large DNA fragments, millions of chromosomes must be sorted which is laborious and time consuming [17]. However, many methods, for example those which utilize PCR to create small amplicons are not constrained by a requirement for large template molecules and can in principle be supported using DNA amplified from sorted chromosomes. Thus, a practical approach to the production of sufficient amounts of moderate-size DNA from particular chromosomes is to sort a more modest number of chromosomes and then amplify their DNA. There are several methods for non-specific DNA amplification, most of them being based on PCR. However, these methods such as DOP-PCR (degenerate oligonucleotide primed PCR) [22] or PEP (primer extension preamplification) [23] are characterized by high amplification bias and provide incomplete genome coverage [24,25]. Moreover, they generate DNA fragments less than 3 kb long, which may be not suitable for some applications.

Recently, a protocol for isothermal multiple displacement amplification (MDA) was developed, which uses the Phi29 polymerase and random primers to amplify the whole genome [24,26]. The protocol has been shown suitable for many applications such as RFLP analysis, chromosome painting [24], comparative genome hybridization [24,27] and SNP genotyping [28-32]. Data obtained from these prior studies indicated that the genome representation achieved after MDA is comprehensive. For example, Paez et al. [30] using high-density oligonucleotide arrays estimated the genome representation to be 99.82% complete. Similarly, Barker et al. [29] observed a concordance of 99.8% in SNP genotyping from genomic DNA and MDA-amplified human DNA, and they achieved a SNP call rate of 98% in both genomic and amplified DNA. Pinard et al. [25] compared two multiple displacement amplification methods, GenomiPhi (GE Healthcare, Chalfont St. Giles, United Kingdom) and Repli-G (Qiagen Sciences Inc., Germantown, USA). In his sequencing-based study, Repli-G generated more amplified DNA, but introduced marginally more bias than GenomiPhi, and generated significantly lower genome coverage, indicating the GenomiPhi the best available system for whole genome amplification.

In this work we have optimized for the first time a protocol for amplification of DNA from flow-sorted plant chromosomes by MDA using barley as our model system. Here we report excellent coverage of amplification, confirmed on a whole genome level using an oligonucleotide pool assay.

## Methods

### Preparation of chromosome suspensions and flow-cytometric sorting

Mitotic metaphase chromosomes of barley (*Hordeum vulgare *L., 2n = 2x = 14) cv. Akcent were flow-sorted according to Lysák et al. [33]. Briefly, barley seedlings were treated subsequently with hydroxyurea and amiprophos-methyl to accumulate meristem root tip cells at metaphase and the synchronized root meristems were fixed by formaldehyde. Chromosome suspensions were prepared by mechanical homogenization of 25 root tips in 1 ml ice-cold LB01 buffer [34] and stained by 2 μg/ml DAPI (4',6-diamidino-2-phenylindole). The stained samples were analyzed using a FACSVantage SE flow cytometer and sorter (Becton Dickinson, San José, USA). Batches of 10,000 chromosomes 1H and of 60,000 chromosomes 2H – 7H were sorted into 50 μl deionized water in a PCR tube. Purity in sorted fractions was checked regularly by FISH using a probe for GAA microsatellite as described in Suchánková et al. [35].

### Purification and amplification of chromosomal DNA

Flow-sorted chromosomes were treated with proteinase K at 50°C for 36 hours in a buffer consisting of 2.5 mM Tris (pH 8.0), 1.25 mM EDTA (pH 8.0) and 0.125% (w/v) SDS. Freshly prepared proteinase K (0.5 mg/ml) was added in a volume of 4 μl to the 10,000-chromosome samples and 8 μl to the 60,000-chromosome samples. Another 2 or 4 μl (half of the original amount) were added after 20 hours of the treatment. The proteinase K was then removed and the buffer was exchanged using Microcon YM-100 column (Millipore Corporation, Bedford, USA) in four rounds of centrifugation at 500 g for 15 min at 23°C. About 450 μl deionized water were added to the column before each centrifugation to wash out the buffer. After purification, the amount of DNA in the samples was estimated using TD-700 fluorometer (Turner Designs, Sunnyvale, USA). As the volume after purification was usually 10–20 μl, it was reduced by overnight evaporation at 4°C to reach volume of 1–2 μl. The amplification of purified chromosomal DNA was performed using GenomiPhi DNA Amplification Kit (GE Healthcare, Chalfont St. Giles, United Kingdom) according to instructions of the manufacturer in a 20 μl reaction for 16 hours. The samples were lyophilized for storage and shipment. For further processing, samples were resuspended in 100 μl of 10 mM Tris-HCl, 0.1 mM EDTA (pH 8.0) of which 50 μl were de-salted on MicroSpin G50 columns (GE Healthcare). Concentrations were measured by absorbance at 260 nm and using the Quant-iT PicoGreen assay (Invitrogen, Carlsbad, USA).

### Analysis of the amplification products by Southern hybridization

Southern hybridization with barley genomic DNA as a probe was used to analyze composition of the amplification product. The probe was labeled using AlkPhos Direct kit (GE Healthcare). In the first experiment, 5 μg of the multiple-displacement-amplified barley chromosomes (1H-7H) were run in a 1.5% agarose gel in 0.5 × TBE and subsequently transferred onto a nylon membrane (Hybond N+, GE Healthcare) by alkali transfer. Hybridization ran overnight at 55°C, followed by standard post-hybridization washes. Visualization of the hybridization product was performed by membrane incubation with a chemiluminiscent substrate (CDP Star, GE Healthcare) followed by 4 hours exposure to X-ray film. To quantify the portion of barley-derived DNA in the amplification product, dot blot analysis was performed. Serial dilutions of 40, 20, 10, 5, 2, 1 and 0.5 ng, respectively, were spotted on a nylon membrane for both barley amplified and unamplified DNA. Hybridization and signal detection were performed as above.

### Real-time quantitative PCR

Real-time quantitative PCR was used to check the purity of the sorted fractions and potential amplification bias of the MDA. Primers were designed for four genes localized on chromosome 1H, and one gene for each of the remaining chromosomes (Table 1). Primer design was done using Primer Express (Applied Biosystems, Foster City, USA). The PCRs were performed in 96-well plates. A standard curve was constructed for each amplicon with serial dilutions of genomic DNA (30, 15, 7.5, 3.75, 1.875, 0.9375, 0.46875 ng/well). Standards and samples were run in duplicate. Each 25 μl reaction consisted of 12.5 μl of SYBR Green PCR Master Mix (Applied Biosystems), 1 μl of each primer (10 μM) and 1.5 μl of DNA (3 ng for the samples and between 30 and 0.46 ng for the standards). The real-time PCR analysis was performed on an Applied Biosystems Prism 7700 Sequence Detection System (Applied Biosystems). Thermocycling was as follows: 50°C for 2 min and 95°C for 10 min followed by 40 cycles of 95°C for 15 s and 60°C for 1 min.

**Table 1:** Primers for real-time quantitative PCR

**Chr.**	**Gene**	**Forward (5' – 3')**	**Reverse (5' – 3')**	**Amplicon (bp)**
1H	*HemA2*	CACGCCATCTGTTTGAGGTATC	TCTTTCCCAGGCCTCCACTAT	120
1H	*Hor3*	TGTGTTGGCAAACTGCACTTG	TTGTGAGGCCCTTAAGTCGG	123
1H	*Hva1*	CTCCACAAGCAGTCGATCCA	GGCCATCTTCGTCTCACGAT	111
1H	*Nbs*	CAACCTACACCGGAAAACTCATCT	TTGTGAGGCCCTTAAGTCGG	137
2H	*Cor14b*	CCCAAACAGGTCACCCAAAG	TGCGTGCGAGACTGTCGAC	139
3H	*Dhn10*	GGGTTCTCGATCTCTTCTTGCAT	TCTTCCTCCGTCCACCCA	177
4H	*Dhn6*	GGACGTACGGCGCTACTGAG	TGGTTCCTCGAGTCTTTATTCTTCA	150
5H	*Dhn9*	GTTCCACGTGATCTTCATTCAATAA	TCAGCAAGAAGACACAAGAACACA	137
6H	*Dhn8*	CCGTCCTTCTTTCTTGCTTGTG	TATAGCCGCTGTCAAGTAGACCG	100
7H	*Ss1*	CTTGGCCGGGATGCGTTA	GGTGTGGGTATCCGAGGGAG	101

### Assessment of amplified genomic DNA using an oligonucleotide pool assay

Genetic marker analysis was done using an Illumina GoldenGate BeadArray (Illumina, San Diego, USA) with an Oligo Pool Assay (OPA) for interrogation of 1524 barley markers [36]. Processing of DNA was done by Joe DeYoung and staff at the Southern California Genotyping Consortium at University of California, Los Angeles, following standard procedures. Marker data were supervised manually using the GenCall software (Illumina). In order to compare the performance of amplified to unamplified genomic DNA, marker analysis included both amplified and unamplified DNA of cv. Akcent, together with unamplified DNA from 102 barley accessions [36]. Marker calls of Akcent were accepted if an Akcent datum clustered with marker data from the germplasm collection. Markers with low GenCall scores were tagged as "no call" and not considered further. To add certainty, three replicates of amplified Akcent DNA were used. The GenCall software's calling function was used to produce genotype allele calls (AA, AB, BB) and the GenCall score. Allele calls from the replicated samples of amplified Akcent and the unamplified sample of Akcent were analyzed for concordance and the reproducibility was evaluated by calculating the coefficient of variation (CV, standard deviation as a percentage of the mean). Only markers with a high concordance and reproducibility were further considered.

### Marker analysis of amplified flow-sorted chromosomes using the OPA

We also analyzed DNA amplified from sorted chromosome 1H and from a pool of sorted chromosomes 2H – 7H. We calculated the ratio of GenCall values from 1H and 2H – 7H for each locus and examined the distribution of these ratios in the context of previously mapped markers to heuristically define 1H, uncertain or 2H – 7H bins.

## Results and Discussion

### Chromosome sorting

**Figure 1:**
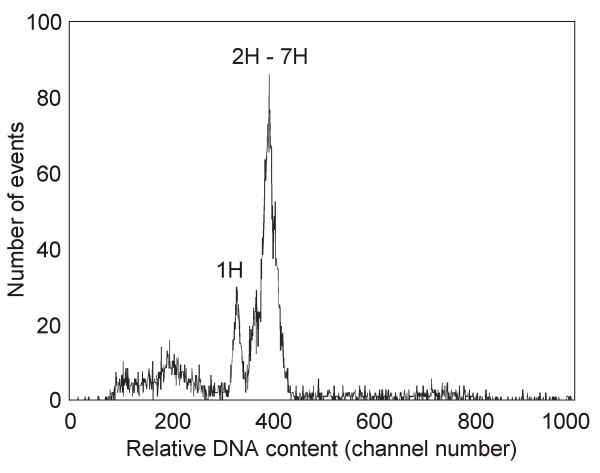
**Flow karyotype of barley**. Histogram of relative DNA contents (flow karyotype) obtained after flow cytometric analysis of DAPI-stained liquid suspension of intact mitotic chromosomes of barley. The peak of chromosome 1H is clearly resolved, which enables isolation of 1H by flow sorting.

Histograms of relative fluorescence intensity obtained after flow-cytometric analysis of chromosomes isolated from barley cv. Akcent consisted of a small peak representing chromosome 1H and a composite peak representing the remaining chromosomes 2H – 7H (Figure 1). This observation confirmed our previous results [33,35] and enabled sorting of chromosome 1H. The purity in sorted 1H fractions as determined by FISH exceeded 95%.

### Optimization of chromosome treatment for DNA amplification

After DNA purification, 7 – 70 ng of chromosomal DNA were obtained from flow-sorted fractions (depending on the number of sorted chromosomes), which were subsequently used for multiple displacement amplification. The yield of amplified DNA varied from 3.7 to 5.7 μg. A critical step of the procedure was the purification of samples after proteinase treatment using the Microcon YM-100 columns. If the buffer was not exchanged completely (by reducing the number of centrifugations), remnants of proteinase K and the buffer interacted with Phi29 polymerase and hampered the amplification. On the other hand, additional rounds of centrifugation and especially spinning to dryness drastically reduced the recovery of chromosomal DNA (to approximately 1 ng). While this did not significantly influence the amount of amplification product, it decreased the representation of some loci in the amplification product as demonstrated by real-time PCR (data not shown). Amplification bias inversely correlating with the amount of template was observed also by Rook et al. [37] and Bergen et al. [38]. These data are in agreement with the manufacturer's instructions according to which at least 1 ng (optimum 10 ng) of purified DNA is to be used for the reaction to warrant minimum amplification bias.

**Figure 2:**
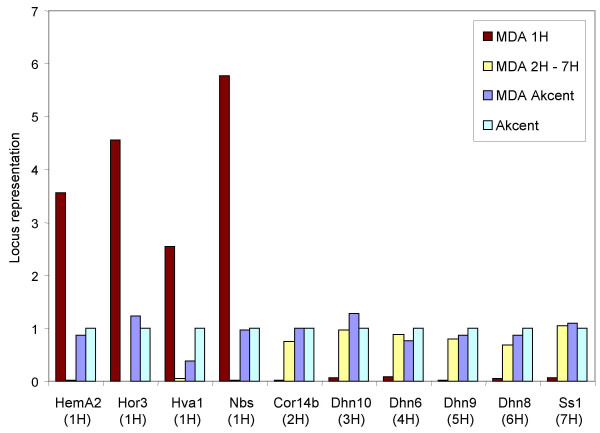
**Locus representation in amplified DNA relative to unamplified DNA**. Four 1H-specific loci and one locus from each of the remaining chromosomes were examined for representation by real-time quantitative PCR with gene-specific primers. The RT PCR was run on amplified 1H chromosomes (MDA 1H), amplified 2H – 7H chromosomes (MDA 2H-7H), amplified Akcent DNA (MDA Akcent) and unamplified Akcent genomic DNA (Akcent).

Quantitative PCR using primers for four genes localized on chromosome 1H (Table 1) showed high quantities of PCR products both in the 1H and genomic DNA samples. Samples of amplified flow-sorted 1H chromosomes showed maximum 2.3-fold difference in the copy number among the 1H-specific loci (Figure 2). Several-fold higher quantities of PCR products obtained with DNA amplified from chromosome 1H reflect the fact that the samples of flow-sorted chromosomes were enriched seven-fold in number of loci per unit of mass as compared to genomic DNA. For genes localized on the remaining chromosomes (2H – 7H) even lower amplification bias (1.5-fold) among the six loci was observed (Figure 2). This is a similar level of amplification bias as observed by Dean et al. [24] who compared amplification of 8 genes from blood and tissue cultured cells and observed less than a three-fold bias, Hosono et al. [28] who analyzed amplification of 47 loci in DNA from clinical samples revealing maximum six-fold bias, and Rook et al. [37] who observed maximum three-fold bias when comparing amplification of 4 loci in samples of laser-capture microdissected cells. Samples of chromosomes 2H – 7H where 60,000 purified chromosomes were used as a template provided lower amplification bias compared with those of 1H where only 10,000 chromosomes were used. This corresponds to similar findings of Rook et al. [37] who observed an inverse correlation between amplification bias and the amount of template. RT-PCR showed only minor contamination of the 1H fraction by other chromosomes (Figure 2).

### Characterization of the amplification product

**Figure 3:**
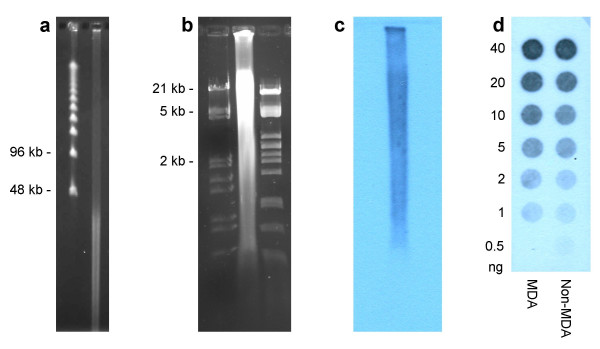
**Analysis of the product of multiple displacement amplification on flow-sorted chromosomes**. (a) 500 ng of the product were analyzed by pulsed field gel electrophoresis in 1% agarose gel, 0.25 × TBE at 12.5°C, 6 V/cm and 5–15 s switch time ramp for 15 hours. Lambda ladder was used as a size standard. (b) 5 μg of the product were analyzed in 1.5% agarose gel in 0.5 × TBE and (c) after transfer on nylon membrane hybridized with barley genomic DNA. (d) Dot blot of barley genomic DNA (Non-MDA) and amplified DNA of all barley chromosomes (MDA) hybridized with barley genomic DNA.

The multiple-displacement-amplified (MDA) DNA of flow-sorted chromosomes was analyzed by electrophoresis under various conditions (Figure 3a, b). The majority of products were found between 5 – 30 kb. The MDA using random hexamers to prime the DNA amplification is known to generate amplification product also in the absence of template DNA [38]. This spurious product is electrophoretically indistinguishable from that obtained in the presence of template. Therefore we analyzed the composition of the MDA product obtained with flow-sorted chromosomes. Southern hybridization with barley genomic DNA used as a probe revealed that the product in its whole size spectrum was derived from barley DNA (Figure 3c). To quantify the portion of barley-derived DNA in the MDA product we performed dot blot analysis comparing serial dilutions of barley amplified and unamplified DNA hybridized with barley genomic DNA. The hybridization showed high concordance of both samples indicating that a large percent of the amplified DNA was barley-derived rather than nonspecific synthesis (Figure 3d).

### Genome wide survey of amplified barley DNA

We previously developed an OPA for interrogation of 1524 barley SNPs [36]. We observed 1426 loci with high-confidence marker calls for Akcent genomic DNA. Comparison of the allele calls for Akcent DNA samples (amplified or unamplified) revealed only one locus with different allele call for one of the amplified replicates compared to un-amplified Akcent DNA, resulting in a concordance of 99.98%. Similar studies using MDA on genomic DNA from human cell lines also resulted in >99% concordance [29,31]. In addition, we found high reproducibility for the three replicates of amplified Akcent DNA; the coefficient of variation (CV) of GenCall scores was below 2% for 1398 of the 1426 loci (98.0%). This shows that the replicates of amplified Akcent DNA clustered very close together as was evident also from manual inspection of the genotyping clusters. In summary, we were able to score the marker call as plus/minus for the entire dataset by including Akcent as a reference dataset.

### Isolated chromosomes as a tool for physical mapping

We also applied the OPA to amplified DNA of flow-sorted barley chromosomes 1H and 2H-7H, respectively (see Materials and Methods). We classified the 1426 loci with good genotype calls from unamplified Akcent genomic DNA into four sub-sets based on their map data: (i) 130 known 1H loci, (ii) 920 known 2H – 7H loci, (iii) 349 loci with previously unknown map position, and (iv) 27 loci with ambiguous map positions (mapped to different chromosomes dependent on the mapping population used).

**Figure 4:**
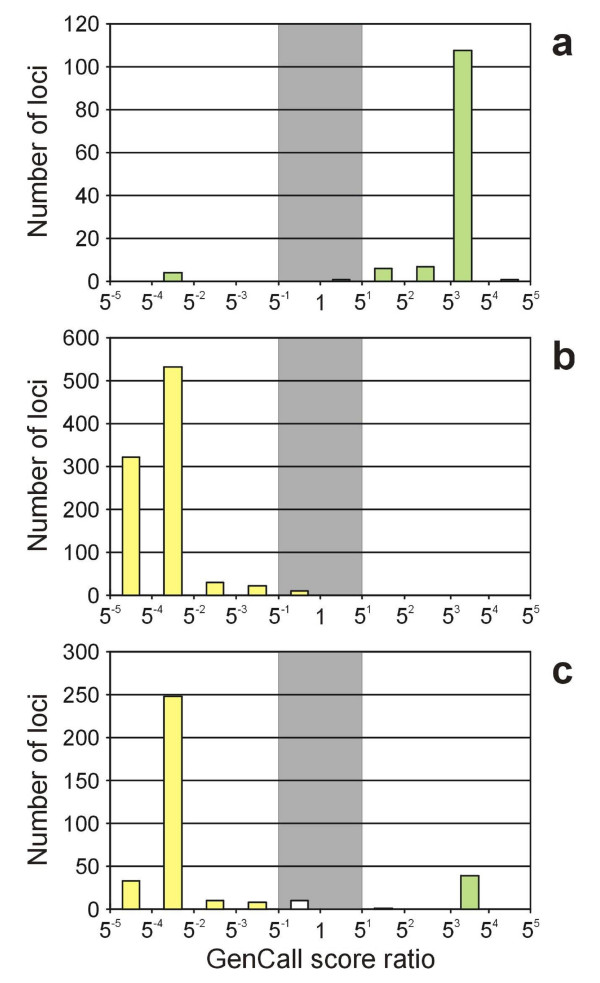
**Histogram of the ratio of GenCall scores (1H/2H-7H)**. (a) 127 loci previously mapped to 1H, (b) 920 loci previously mapped to 2H-7H and (c) mapping 349 loci with unknown map position. Loci mapped to 1H (GenCall score ratio 5^1 ^– 5^5^) are shown in green. Loci mapped to 2H – 7H (GenCall score ratio 5^-5 ^– 5^-1^) are shown in yellow. Cutoff region (GenCall score ratio 5^-1 ^– 5) is shown in grey. Note that the X axis is shown in log_5 _scale.

For the 130 loci previously mapped to 1H, we expected a high GenCall 1H/2H-7H score ratio (see Materials and Methods). This expectation was fulfilled for 93.8% of these markers (122 of 130) which had a ratio of at least 5.0 (Figure 4a). Interestingly, four loci were nulls in the 1H fraction but not in the 2H – 7H fraction (these loci had a ratio of 0.003 – 0.002). These anomalies have been subsequently explained as an incorrect assumption that sub-clustering patterns within the genotyping data represented the targeted SNP.

We conducted a parallel analysis of the 920 loci previously mapped to chromosomes 2H – 7H, of which the GenCall score ratios for 98.7% (908 of 920) were less than 0.2 (Figure 4b). One locus had a ratio of 162, which also has subsequently been explained as incorrect use of sub-clusters to represent SNP variation in the targeted marker position.

Based on the above two analyses of 1H and 2H – 7H markers and selecting ratios of 5.0 for 1H and 0.2 for 2H – 7H, we can conclude that only 20 out of 1050 previously mapped loci (1.9%) failed to be allocated to one of these two marker sets.

### Associating markers with unknown map position to chromosome 1H

A total of 349 loci targeted by the OPA and yielding high quality Akcent data were not previously mapped. The GenCall score ratio cutoff values discussed above removed 10 loci from further consideration and partitioned the remaining 339 loci into two bins: the 1H bin contained 40 markers (11.8%) and the 2H – 7H bin contained 299 markers (88.2%) (Figure 4c). These proportions are very near the expected proportion of slightly less than 1/7 of the total barley genome contained in chromosome 1H. We analyzed barley-rice synteny by BLASTX of all rice proteins against these 40 HarvEST:Barley unigenes mapped to 1H [see Additional file 1]. The rice chromosomes with highest number of best blast hits were chromosomes five and ten with 24 and 8 hits, respectively. Stein et al. [39] placed 93 markers on 1H and also found the highest degree of synteny to rice chromosome five followed by chromosome ten.

### Genotyping of isolated chromosomes to clarify the map location of ambiguous loci

The consensus map used in [36] contained 1153 OPA-based loci, of which 27 markers mapped to 2 different chromosomes (a total of 54 ambiguous markers). Of these 54 markers, 14 were mapped to 1H in at least one mapping population and had a high quality genotype call using Akcent DNA. In order to solve the correct map position for these ambiguous loci we examined the GenCall ratios as discussed above (Table 2). The correct chromosome assignments for 12 of these 14 loci were readily apparent from the GenCall ratios. The remaining two loci encode a GTP binding protein and a chlorophyll A/B binding protein, both of which belong to large multigene families, with 58 and 40 members, respectively. The high GenCall scores for these loci both in 1H and 2H – 7H fractions indicate that the SNP markers target two loci on different chromosomes.

**Table 2:** Using sorted chromosomes 1H and 2H – 7H to determine chromosome location of ambiguously mapped loci

**Locus name**	**Morex × Barke**	**OWBs**	**Steptoe × Morex**	**Allele call Akcent**	**Allele call 1H**	**Allele call 2H-7H**	**Ratio of GenCall score**^**a**^	**Chrom.**^**b**^
1_0198	1H	1H	5H	B	B	B	488	1H
1_0716	1H	3H	3H	A	A	A	479	1H
1_0549	6H	Nd	1H	B	B	B	436	1H
1_0443	1H	Nd	5H	B	B	AB	435	1H
1_0644	1H	6H	nd	B	B	B	412	1H
1_0075	1H	7H	7H	A	A	B	306	1H
1_1223	1H	5H	nd	B	B	AB	299	1H
1_0148	nd	1H	4H	A	A	AB	252	1H
1_0059	3H	1H	nd	A	A	B	1	nd
1_0828	6H	1H	nd	A	B	A	1	nd
1_0942	nd	4H	1H	B	B	B	1/476	4H
1_0316	1H	7H	7H	B	B	B	1/488	7H
1_1092	5H	1H	5H	B	B	B	1/515	5H
1_1100	nd	2H	1H	B	B	B	1/722	2H

nd – not determined, ^a^1H GenCall score/2H-7H GenCall score, ^b^chromosome location as determined by comparing GenCall scores for 1H and 2H – 7H

To summarize, among the 1426 interrogated Akcent loci, 1381 were unambiguously allocated to 1H or 2H-7H using flow-sorted chromosome DNA [see Additional file 2].

## Conclusion

The present study demonstrates a method to produce unbiased microgram quantities of DNA from a small number of flow-sorted plant chromosomes, suitable for high throughput genetic marker systems. This is a significant breakthrough as the preparation of chromosomes in microgram quantities DNA requires weeks of sorting [17]. Depending on chromosome size, the ten ng quantity, required for representative amplification translates to only about 10 – 20 thousand chromosomes. With a sorting speed of 20 chromosomes/sec [2], the required number of chromosomes can be sorted in less than 30 minutes.

Although the amplified DNA of flow-sorted chromosomes is not of high molecular weight, the size of the amplified fragments (5 – 30 kb) may suit various applications, including the construction of chromosome-specific short-insert DNA libraries and genotyping assays. Short-insert chromosome-specific DNA libraries constructed after MDA facilitated development of molecular markers from particular genome regions [11].

The use of the novel approach presented here is not limited to barley as the chromosome sorting technology has been developed for 17 plant species, including tetraploid and hexaploid wheat, rye or several legume species [6]. In some species it is possible to sort single chromosome arms, which represent only a few percent of the whole genome [8,35]. The ability to dissect genomes to small fractions is especially attractive when dealing with complex plant genomes.

We have established that chromosome sorting coupled with DNA amplification and the Illumina GoldenGate assay provides a powerful approach towards parallel mapping of DNA sequences to particular chromosomes. In this work, we have mapped 162 SNP loci to chromosome 1H, including 40 loci with hitherto unknown map position. As the position of markers on genetic maps often can be questioned, we expect that this approach can be used to clarify many ambiguities. In summary, the ability to rapidly produce micrograms of chromosome-specific DNA significantly broadens the range of applications of flow-sorted chromosomes and chromosome arms in plant genomics.

## Abbreviations

BAC: Bacterial Artificial Chromosome; CV: coefficient of variation; DOP-PCR: degenerate oligonucleotide primed PCR; FISH: fluorescence *in situ *hybridization; MDA: multiple displacement amplification; PEP: primer extension preamplification; PRINS: primed *in situ *labeling; RFLP: restriction fragment length polymorphism; SNP: single nucleotide polymorphism.

## Authors' contributions

HŠ and JB optimized the procedure for multiple displacement amplification of flow-sorted chromosomes. JTS and PC performed real-time PCR analysis of amplification products. JTS analyzed the genotyping data and developed the strategy for bin mapping. EH analyzed the amplification product by Southern hybridization. PS flow sorted the barley chromosomes. PRB and TJC examined and helped resolve map position ambiguities. JŠ made an intellectual contribution to the concept of the experiment. HŠ and JTS drafted the manuscript. JD and TJC conceived and supervised the project and prepared the final version of the manuscript.

## Supplementary Material

Additional file 1 **Analysis of 40 HarvEST:Barley unigenes mapped to 1H**. XLS file with a table displaying results of analysis of barley-rice synteny based on BLASTX of all rice proteins against these 40 HarvEST:Barley unigenes mapped to 1H. Click here for file

Additional file 2 **Scheme of the mapping experiment**. PDF file with a chart displaying illustratively the process of mapping using flow-sorted chromosomes and the results obtained. In the first stage, 1050 loci with known map position were analyzed (a) to define selecting GenCall score ratios for bin mapping. These parameters were used to analyze 349 loci with unknown map position (b) and to clarify the map location of 14 ambiguous loci (c). Click here for file
