# Supplementary figures and images for: Coupling amplified DNA from flow-sorted chromosomes to high-density SNP mapping in barley
Source: BMC Genomics. 2008 Jun 19;9:294. doi: 10.1186/1471-2164-9-294 (PMC2453526; doi:10.1186/1471-2164-9-294)

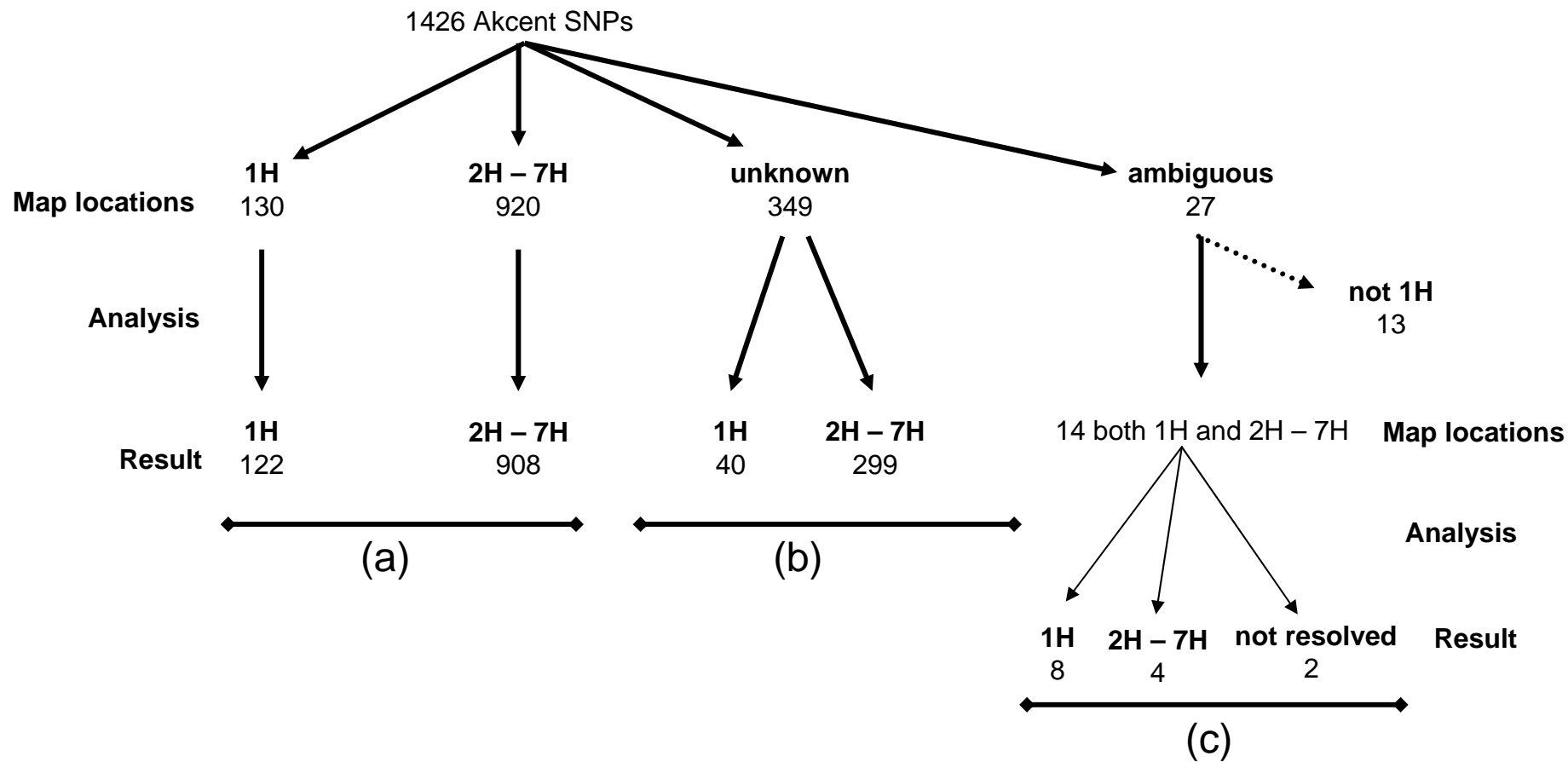

Supplemental File 2. Scheme of the mapping experiment

Supplement: Additional file 2 — Scheme of the mapping experiment. PDF file with a chart displaying illustratively the process of mapping using flow-sorted chromosomes and the results obtained. In the first stage, 1050 loci with known map position were analyzed (a) to define selecting GenCall score ratios for bin mapping. These parameters were used to analyze 349 loci with unknown map position (b) and to clarify the map location of 14 ambiguous loci (c). [file 1471-2164-9-294-S2.pdf]
